# NAC transcription factor JUNGBRUNNEN1 enhances drought tolerance in tomato

**DOI:** 10.1111/pbi.12776

**Published:** 2017-08-04

**Authors:** Venkatesh P. Thirumalaikumar, Vikas Devkar, Nikolay Mehterov, Shawkat Ali, Rengin Ozgur, Ismail Turkan, Bernd Mueller‐Roeber, Salma Balazadeh

**Affiliations:** ^1^ Institute of Biochemistry and Biology University of Potsdam Potsdam‐Golm Germany; ^2^ Max Planck Institute of Molecular Plant Physiology Potsdam‐Golm Germany; ^3^ Division of Biological and Environmental Sciences and Engineering Center for Desert Agriculture King Abdullah University of Science and Technology (KAUST) Thuwal Saudi Arabia; ^4^ Department of Biology Faculty of Science Ege University Izmir Turkey; ^5^ Present address: Department of Medical Biology Medical University of Plovdiv BG ‐ 4000 Plovdiv Bulgaria

**Keywords:** *Arabidopsis*, tomato, transcription factor, drought, reactive oxygen species, DELLA

## Abstract

Water deficit (drought stress) massively restricts plant growth and the yield of crops; reducing the deleterious effects of drought is therefore of high agricultural relevance. Drought triggers diverse cellular processes including the inhibition of photosynthesis, the accumulation of cell‐damaging reactive oxygen species and gene expression reprogramming, besides others. Transcription factors (TF) are central regulators of transcriptional reprogramming and expression of many TF genes is affected by drought, including members of the NAC family. Here, we identify the NAC factor JUNGBRUNNEN1 (JUB1) as a regulator of drought tolerance in tomato (*Solanum lycopersicum*). Expression of tomato *JUB1* (*SlJUB1*) is enhanced by various abiotic stresses, including drought. Inhibiting *SlJUB1* by virus‐induced gene silencing drastically lowers drought tolerance concomitant with an increase in ion leakage, an elevation of hydrogen peroxide (H_2_O_2_) levels and a decrease in the expression of various drought‐responsive genes. In contrast, overexpression of *AtJUB1* from *Arabidopsis thaliana* increases drought tolerance in tomato, alongside with a higher relative leaf water content during drought and reduced H_2_O_2_ levels. AtJUB1 was previously shown to stimulate expression of *DREB2A*, a TF involved in drought responses, and of the DELLA genes *GAI* and *RGL1*. We show here that SlJUB1 similarly controls the expression of the tomato orthologs *SlDREB1*,* SlDREB2* and *SlDELLA*. Furthermore, AtJUB1 directly binds to the promoters of *SlDREB1*,* SlDREB2* and *SlDELLA* in tomato. Our study highlights JUB1 as a transcriptional regulator of drought tolerance and suggests considerable conservation of the abiotic stress‐related gene regulatory networks controlled by this NAC factor between Arabidopsis and tomato.

## Introduction

Water deficit (drought) represents one of the most significant abiotic stresses limiting plant growth, development and productivity. Drought triggers several responses in plants including a cessation of shoot growth*,* the inhibition of the initiation of new leaves and the promotion of senescence in older leaves leading to a remarkable decrease in canopy size and crop yield (Degenkolbe *et al*., 2009; Harris *et al*., 2007; Martínez *et al*., 2007; Rivero *et al*., 2007). At the cellular level, drought stress triggers an excessive generation of reactive oxygen species (ROS), thereby affecting redox homeostasis and resulting in oxidative stress as evidenced by a decline in photosynthetic efficiency, severe cellular damage by peroxidation, reduced cell membrane stability, increased protein denaturation and leaf wilting (Benjamin and Nielsen, 2006; Choudhury *et al*., 2013, 2016; Cruz de Carvalho, 2008; Hanin *et al*., 2011).

As sessile organisms, plants have evolved impressive strategies at molecular, biochemical, physiological and developmental levels to cope with, and adapt to, water deficit (Basu *et al*., 2016; Lata and Prasad, 2011; Li *et al*., 2014; Tamura *et al*., 2003). The coordinated regulation of gene expression represents one such sophisticated response to drought stress. Water deficit triggers a wide‐scale reprogramming of the transcriptome whereby transcription factors (TFs), and the gene regulatory networks (GRNs) they control, are of central importance (Chen *et al*., 2016; Joshi *et al*., 2016; Rabara *et al*., 2014; Todaka *et al*., 2015; Vermeirssen *et al*., 2014).

NAC (NAM, ATAF and CUC) transcription factors are widespread in plants and the expression of many *NAC* genes is induced by abiotic and biotic stresses (Nakashima *et al*., 2012; Nuruzzaman *et al*., 2013; Shao *et al*., 2015).

Over the last decade, various NAC TFs in different plant species, including crops, have been shown to be suitable tools for the improvement of plant responses to dehydration/drought stress. For example, in *Arabidopsis thaliana*, transgenic plants overexpressing *ANAC019*,* ANAC055* or *ANAC072*/*RD26* exhibit an enhanced expression of stress‐responsive genes and an improved tolerance to drought and salinity stress (Tran *et al*., 2004). All three NAC TFs interact with ZINC FINGER HOMEODOMAIN1 (ZFHD1), a TF transcriptionally induced by the phytohormone abscisic acid (ABA), drought and high salinity, bind to the promoter of *EARLY RESPONSIVE TO DEHYDRATION STRESS1 (ERD1)* and regulate the response to drought (Fujita *et al*., 2004; Nakashima *et al*., 1997; Tran *et al*., 2004, 2007). In addition, ANAC016 has recently been reported as a positive regulator of the plant′s response to drought stress in Arabidopsis (Sakuraba *et al*., 2015). Mutants lacking functional ANAC016 show a high tolerance to drought, while *ANAC016* overexpressors are sensitive to drought and display accelerated senescence. ANAC016 suppresses the expression of *ABA‐RESPONSIVE ELEMENT‐BINDING PROTEIN 1* (*AREB1*), a negative regulator of ABA signalling, but activates the expression of *AtNAP*, a NAC transcription factor mediating drought response by negatively regulating ABA signalling (Sakuraba *et al*., 2015; Zhang and Gan, 2012). In rice, overexpression of both *SNAC3* (*ONAC003*) and *OsNAC6* resulted in improved drought tolerance in transgenic plants (Fang *et al*., 2015; Nakashima *et al*., 2007). In barley (*Hordeum vulgare*), expression of the *HvSNAC1* gene is induced by multiple stresses and transgenic plants overexpressing *HvSNAC1* show improved drought tolerance (Al Abdallat *et al*., 2014).

Tomato (*Solanum lycopersicum* L.) is an important vegetable fruit crop grown globally. Most tomato cultivars are susceptible to abiotic stresses such as drought. Under drought conditions, the growth of tomato plants is inhibited and fruit yield is significantly reduced (Foolad *et al*., 2003; Landi *et al*., 2016; Nuruddin *et al*., 2003; Qi *et al*., 2016). Therefore, the identification of genetic determinants of drought stress tolerance in tomato is an important task for agricultural development. Several NAC transcription factors are transcriptionally induced by drought in tomato, but only a few of them have been functionally characterized so far (Han *et al*., 2012; Liu *et al*., 2014; Wang *et al*., 2016; Zhu *et al*., 2014a). It has been shown that *SINAC4*, a MeJA (but not ABA)‐induced NAC, positively regulates the response of tomato plants to salt and drought stress (Zhu *et al*., 2014a). Transgenic *SINAC4‐RNAi* lines showed reduced tolerance to drought (and salt stress) and a reduced expression of stress‐related genes (Zhu *et al*., 2014a). SlNAC35 is another NAC TF from tomato that positively regulates the response to drought when overexpressed in tobacco (*Nicotiana tabacum*). *SlNAC35* is a homologue of *AtNAP* from Arabidopsis. It has been shown recently that overexpression of *SlNAC35* in tobacco results in better root growth and development under drought and salt stresses by affecting auxin signalling and the expression of several *AUXIN RESPONSE FACTOR* (*ARF*) genes (Wang *et al*., 2016). In contrast, SlSRN1 (*Solanum lycopersicum* stress‐related NAC1) appeared to be a negative regulator of oxidative and drought stress responses (Liu *et al*., 2014). Although the above‐mentioned NACs have been shown to regulate the response to drought stress in tomato, the underlying molecular mechanisms and stress‐related genes directly regulated by them are largely unknown.

In this study, we investigated the function of the NAC transcription factor JUNGBRUNNEN1 (JUB1) for the response of tomato to drought stress. Previously, we showed that JUB1 (ANAC042) from *Arabidopsis thaliana* (hereafter, AtJUB1) functions as a central regulator of plant longevity and the interplay between growth and stress responses (Shahnejat‐Bushehri *et al*., 2016; Wu *et al*., 2012). We reported that AtJUB1 exerts its role in controlling the response to stress in part through dampening cellular hydrogen peroxide (H_2_O_2_) level. Notably, the intracellular level of H_2_O_2_ is significantly reduced in *JUB1* overexpressors but enhanced in *jub1‐1* knockdown plants (Wu *et al*., 2012). AtJUB1 mediates the interplay between ROS and stress responses by regulating functionally diverse target genes. For example, AtJUB1 directly activates expression of *DREB2A* (*DEHYDRATION‐RESPONSIVE ELEMENT‐BINDING PROTEIN 2A*) which encodes an AP2‐type TF involved in the regulation of drought and heat responses (Kant *et al*., 2008; Sakuma *et al*., 2006). In a transcription factor control cascade, DREB2A is an upstream regulator of *Heat‐shock factor A2* (*HsfA2*) and thereby several *HEAT‐SHOCK PROTEIN* (*HSP*) genes and genes encoding H_2_O_2_ scavenging enzymes (Schramm *et al*., 2008; Yoshida *et al*., 2008). Furthermore, AtJUB1 directly represses the expression of genes encoding key enzymes of gibberellic acid (GA) and brassinosteroid (BR) biosynthesis; this reduces the levels of both growth hormones, thereby leading to the stabilization of DELLA proteins (Shahnejat‐Bushehri *et al*., 2016). Additionally, AtJUB1 directly binds to the promoters of *DELLA* (*GAI* and *RGL1*) genes and positively regulates their expression. DELLA proteins belong to the GRAS family of transcriptional regulators and are known as master repressors of growth. Moreover, accumulation of DELLA proteins promotes stress tolerance by restraining stress‐induced ROS accumulation (Achard *et al*., 2008a,b). The critical role of JUB1 in restraining ROS accumulation holds great promise for this TF as a candidate for genetic engineering of improved drought responses in crops.

Here, we provide compelling evidence that JUB1 positively regulates the tolerance of tomato plants to drought. We demonstrate that tomato plants with reduced expression of *SlJUB1* (*Solanum lycopersicum JUB1*;* Solyc05G021090*), the closest homologue to Arabidopsis *AtJUB1*, are more sensitive to drought than control plants and exhibit a higher level of oxidative stress. In contrast, transgenic tomatoes ectopically expressing *AtJUB1* are more tolerant to stress and show reduced oxidative damage. Furthermore, we identified *SlDREB1, SlDREB2* and *SlDELLA* as potential direct target genes of SlJUB1 during drought stress. This study highlights the role of the SlJUB1 transcription factor as a regulator of drought tolerance in tomato and suggests considerable conservation of the abiotic stress‐related gene regulatory network (GRN) controlled by JUB1 between *Arabidopsis* and tomato.

## Results

### Functional analysis of *SlJUB1* in tomato

To study the role of JUB1 for the regulation of drought in tomato, we first investigated the tomato genome for the presence of *JUB1* gene(s) using the Sol Genomics database (https://solgenomics.net/) employing the BLASTP algorithm. *Solyc05G021090* (hereafter, *SlJUB1*) was identified as the closest homologue to AtJUB1 (62.6% similarity at the amino acid level). An amino acid sequence alignment of SlJUB1 with AtJUB1 and other known NAC proteins from tomato including SlNAC1 (Selth *et al*., 2005), SlNAC2 (Uppalapati *et al*., 2008), SlNAC3 (Han *et al*., 2012) and SlNAM (Blein *et al*., 2008) shows the presence of the conserved motifs A to E typical for the DNA‐binding domain of NAC transcription factors (Zhu *et al*., 2014b; Figure S1).

**Figure 1:**
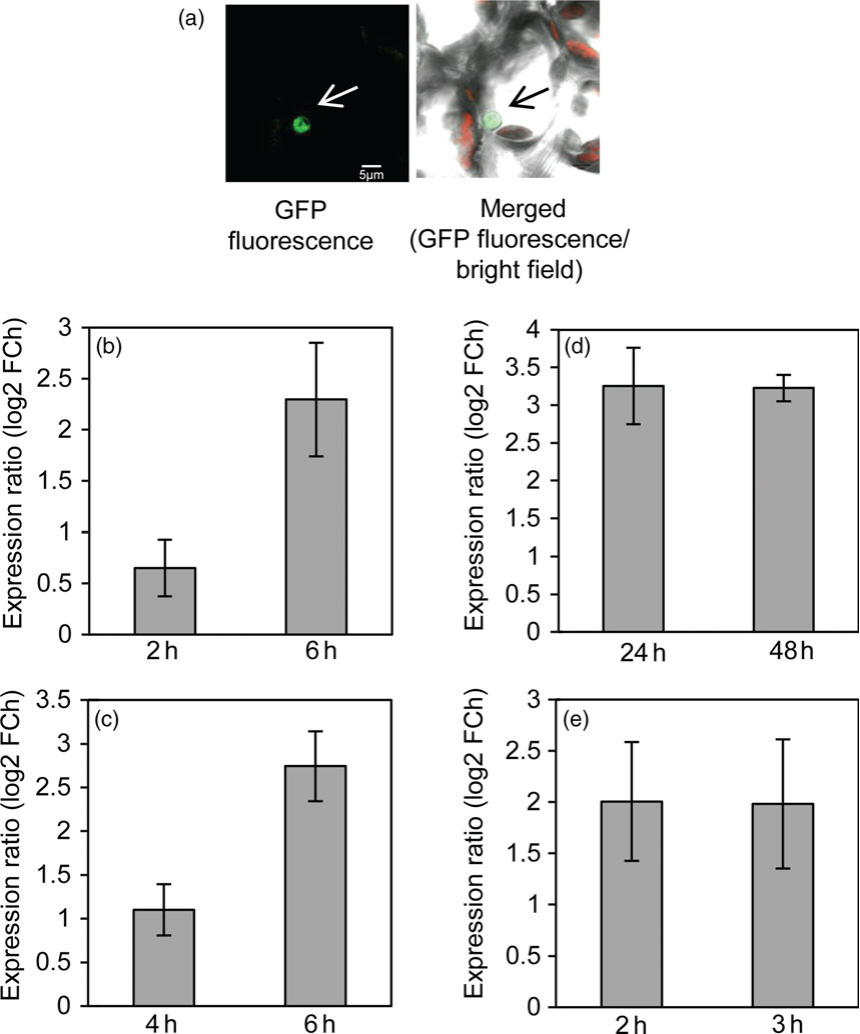
*SlJUB1* encodes a nuclear protein and is induced by various abiotic stresses. (a) Confocal microscope image showing nuclear localization of SlJUB1‐GFP fusion protein upon transient expression in *N. benthamiana* leaf cells. Scale bar, 5 μm. (b–d) *SlJUB1* expression upon treatment with (b) H_2_O_2_ (5 mm), (c) NaCl (200 mm), (d) PEG 6000 (20% [w/v]). Three‐week‐old tomato seedlings were subjected to the stress treatments and harvested at the time points indicated at the x‐axes. (e) *SlJUB1* expression upon dehydration treatment. Terminal leaflets (of leaf 2) were detached and subjected to 2 h and 3 h of desiccation, respectively. Transcript levels were measured using qRT‐PCR; numbers at the *y*‐axis indicate fold change (FCh; log2 basis) compared to controls (unstressed plants). Data represent means ± SD (two independent biological replications with three technical replications per assay).

To investigate the subcellular localization of SlJUB1, *Nicotiana benthamiana* leaves were transiently transformed with a *35S:SlJUB1‐GFP* construct. Analysis using confocal microscope revealed strong GFP fluorescence in the nucleus, in accordance with the function of SlJUB1 as a TF (Figure 1a).

To test whether *SlJUB1* expression is affected by abiotic stresses, we subjected tomato (*Solanum lycopersicum* cv. Moneymaker) plants to various treatments and determined *SlJUB1* expression by quantitative real‐time polymerase chain reaction (qRT‐PCR). Three‐week‐old tomato seedlings were subjected to H_2_O_2_ (5 mm) for 2 h and 6 h, polyethylene glycol (PEG) 6000 (20% [w/v]) for 1 and 2 days and salinity treatment (200 mm NaCl) for 4 h and 6 h and harvested for gene expression analysis. As shown in Figure 1b–d, expression of *SlJUB1* was induced upon all these treatments. Furthermore, we subjected mature leaves (terminal leaflets of leaf number 2) to 2 h and 3 h of desiccation and analysed *SlJUB1* expression. Expression of *SlJUB1* was enhanced at both time points (Figure 1e) raising the possibility that SlJUB1 is involved in drought signalling.

### Silencing of *SlJUB1* results in reduced tolerance to water deprivation

To elucidate the possible involvement of SlJUB1 in the response to drought, we performed virus‐induced gene silencing (VIGS) using a tobacco rattle virus (TRV)‐based system (Liu *et al*., 2002) to reduce *SlJUB1* mRNA levels in tomato leaves. To this end, tomato seedlings were infected with *pTRV1* and recombinant *pTRV2* constructs containing *SlJUB1* and *GUS* (as control).

**Figure 2:**
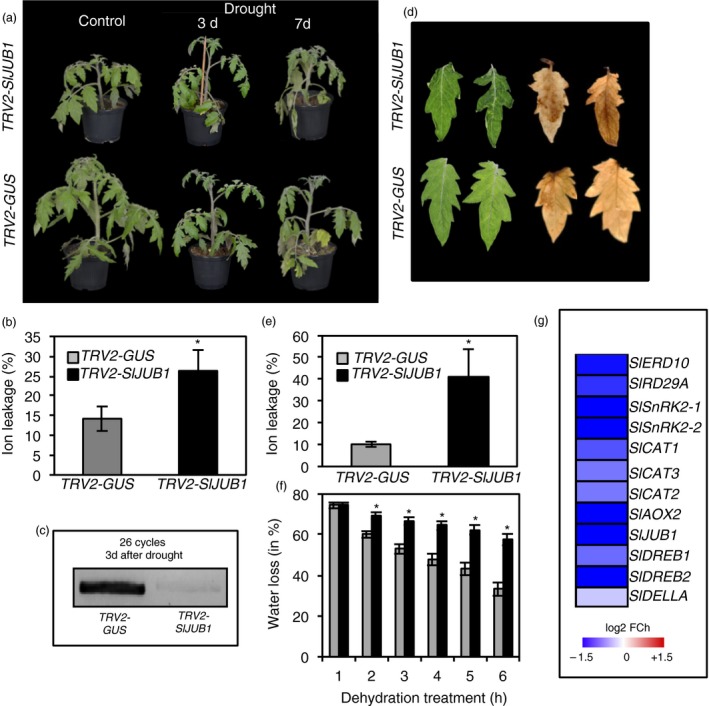
Suppression of *SlJUB1* leads to drought sensitivity in tomato. The role of *SlJUB1* for drought sensitivity was assessed by VIGS. (a) Phenotypes of *TRV2‐SlJUB1* and *TRV2‐GUS* (control) plants under control condition (well watered; left) and after drought stress (3 days: middle; 7 days: right). Note the more severe leaf wilting in the *TRV2‐SlJUB1* plant. (b) Ion leakage of *TRV2‐GUS* and *TRV2‐SlJUB1* leaves (leaf no. 2, terminal leaflet) 7 days after start of the drought treatment. Data represent means ± SD (*n* = 3). (c) Endpoint PCR analysis of *SlJUB1* expression in *TRV2‐GUS* and *TRV2‐SlJUB1* plants after 3 days of drought stress. (d) Phenotypes of detached terminal leaflets from leaf no. 2 (left) and DAB staining for visualization of ROS accumulation (right) of *TRV2‐SlJUB1* (upper row) and *pTRV2‐GUS* plants (lower row) subjected to dehydration treatment for 3 h. (e) Ion leakage of *TRV2‐GUS* and *TRV2‐SlJUB1* leaves after 10 h of dehydration treatment. (f) Water loss in detached leaves of *TRV2‐GUS* (grey columns) and *TRV2‐SlJUB1* (black columns) plants. Data represent means ± SD (*n* = 3). Asterisk in panels (b), (e) and (f) represent statistically significant differences between *TRV2‐SlJUB1* and *TRV2‐GUS* (Student's *t*‐test; *P *<* *0.05). (g) Heatmap showing the fold change (log2 basis) difference in the expression of drought‐responsive genes and tomato orthologs of AtJUB1 direct target genes, compared between *TRV2‐SlJUB1* and *TRV2‐GUS* plants after drought stress (2 h). Gene expression was determined by qRT‐PCR. Data represent the mean of two biological replications with three technical replications per assay.

*SlJUB1‐*silenced and control plants (hereafter, *TRV2‐SlJUB1* and *TRV2‐GUS,* respectively) were then subjected to drought stress by withholding water. As shown in Figure 2a, *TRV2‐SlJUB1* plants started to show some leaf wilting phenotype already after 3 days of drought and the phenotype became more severe after 7 days of drought when compared to the control plants. Electrolyte leakage measurements performed after 7 days of drought revealed a higher membrane damage in *TRV2‐SlJUB1* than in *TRV2‐GUS* plants (Figure 2b). We also measured the transcript levels of *SlJUB1* to verify specificity of the VIGS constructs. Transcript accumulation of *SlJUB1* was significantly reduced in *TRV2‐SlJUB1* plants compared to *TRV2‐GUS* plants during drought stress (Figure 2c). Next, fully expanded leaves (terminal leaflet of leaf number 2) of 3‐week‐old *TRV2‐SlJUB1* and *TRV2‐GUS* plants were detached and subjected to desiccation. As shown in Figure 2d, leaves of *TRV2‐SlJUB1* plants exhibited severe wilting symptoms after dehydration of 3 h. The cellular level of H_2_O_2_ detected by DAB staining was higher in *SlJUB1*‐silenced leaves (3 h after dehydration) than in *pTRV2‐GUS* leaves (Figure 2d). Accordingly, a higher ion leakage due to enhanced membrane damage was observed in leaves of *pTRV2‐SlJUB1* than *pTRV2‐GUS* plants (Figure 2e). Water loss in terminal leaflets (leaf 2) analysed over a 6‐h period was significantly higher in *SlJUB1‐*silenced plants than control plants (Figure 2f).

**Figure 3:**
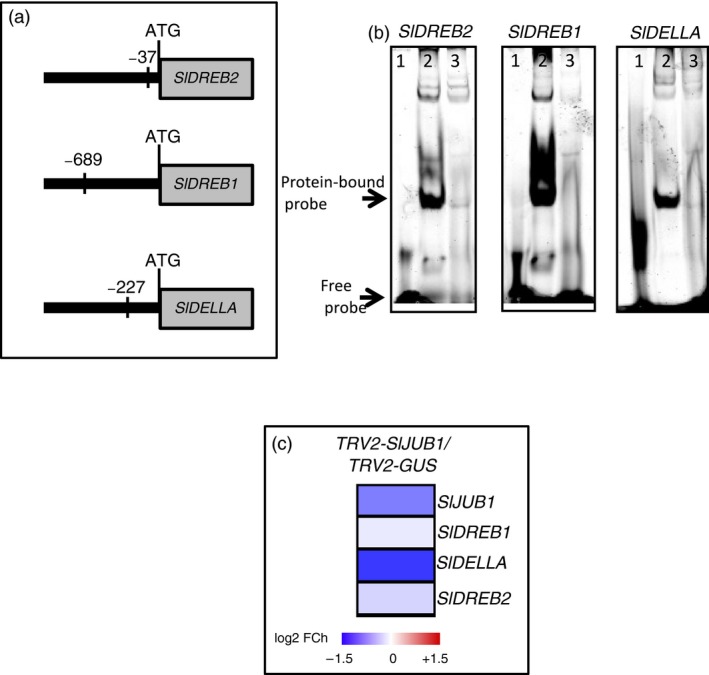
SlJUB1 binds to the promoters of *SlDREB2, SlDREB1* and *SlDELLA*. (a) Schematic representation of the position of the AtJUB1 binding sites in the promoters of *SlDREB2, SlDREB1* and *SlDELLA* (relative to the translation start codon; numbers indicate the start position of the binding sites). Binding sites are located on the forward strand in the case of *SlDREB2* and *SlDELLA*, and on the reverse strand in the case of *SlDREB1*. (b) EMSA showing binding of SlJUB1 to *SlDREB2, SlDREB1* and *SlDELLA* promoter regions harbouring the JUB1 binding site; 1, labelled probe (5′‐DY682‐labelled double‐stranded oligonucleotide) only; 2, labelled probe plus SlJUB1‐GST protein; 3, labelled probe, SlJUB1‐GST protein and 100× competitor DNA (unlabelled oligonucleotide containing SlJUB1 binding site). (c) Transcript levels of *SlJUB1, SlDREB1, SlDELLA* and *SlDREB2* in *TRV2‐SlJUB1* plants 3 days after water withholding compared with *TRV2‐GUS*. Expression was analysed by qRT‐PCR. Data represent the means of three independent experiments.

To reveal the molecular mechanism through which SlJUB1 exerts its role in the response to drought, we compared the expression of tomato orthologs of Arabidopsis drought‐responsive genes as well as of orthologs of Arabidopsis genes that are direct targets of AtJUB1 (*DREB2A, GAI, GA3ox1* and *DWF4*) in *TRV2‐SlJUB1* and *TRV2‐GUS* plants at 2 h of dehydration. Our results revealed that expression of several drought‐responsive genes was reduced in leaves of *TRV2‐SlJUB1* compared to *TRV2‐GUS* plants (Figure 2g). Among the tomato orthologs of AtJUB1 target genes, expression of *SlDREB1* (*Solyc06g050520*), *SlDREB2* (*Solyc05g052410*) and *SlDELLA (Solyc11g011260)* was reduced in *TRV2‐SlJUB1* compared to *TRV2‐GUS* upon dehydration. We next searched 1‐kb promoter regions of the down‐regulated genes for the presence of the core JUB1 binding site (based on knowledge from Arabidopsis; Wu *et al*., 2012). Among those, *SlDREB1, SlDREB2* and *SlDELLA* harbour the AtJUB1 binding site in the promoter regions (Figure 3a) raising the possibility of direct interactions. We next employed electrophoretic mobility shift assays (EMSA) to test for physical interaction of SlJUB1 with the respective promoter sequences of *SlDREB1*,* SlDREB2* and *SlDELLA*. Retardation bands seen in Figure 3b indicate that SlJUB1 specifically interacts with the promoter sequences of all three genes. This interaction is significantly reduced when unlabelled promoter fragments (competitors) are added in excess. Expression of *SlDREB1*,* SlDREB2* and *SIDELLA* was also significantly reduced in *TRV2‐SlJUB1* compared to *TRV2‐GUS* when the plants were subjected to water withholding for 3 days (Figure 3c). Collectively, our data suggest that SlJUB1 is a regulator of the response to drought stress in tomato acting upstream of *SlDREB1*,* SlDREB2* and *SlDELLA*.

### Tomato plants ectopically expressing *AtJUB1* are more tolerant to drought

To further investigate the association of *JUB1* with drought tolerance in tomato, we analysed the phenotypes of tomato plants ectopically expressing *AtJUB1*. These plants were generated by transforming a DNA cassette containing the coding sequence of *AtJUB1* fused to *GFP*, driven by the cauliflower mosaic virus (CaMV) 35S promoter, into the tomato genome (*Solanum lycopersicum* cv. Moneymaker). Different transgenic lines with high and moderate expression of *AtJUB1* were obtained (Shahnejat‐Bushehri *et al*., 2017). Transgenic tomato lines with high levels of *AtJUB1* expression revealed growth‐restricted phenotypes associated with GA and BR deficiencies (such as smaller shoots, smaller leaves and short petioles), similar to *Arabidopsis AtJUB1* overexpressors (Shahnejat‐Bushehri *et al*., 2016, 2017), while the lines with moderate expression of *AtJUB1* (hereafter, *OX1* and *OX3*) showed marginal differences in growth and morphology compared to wild‐type (MM) plants.

**Figure 4:**
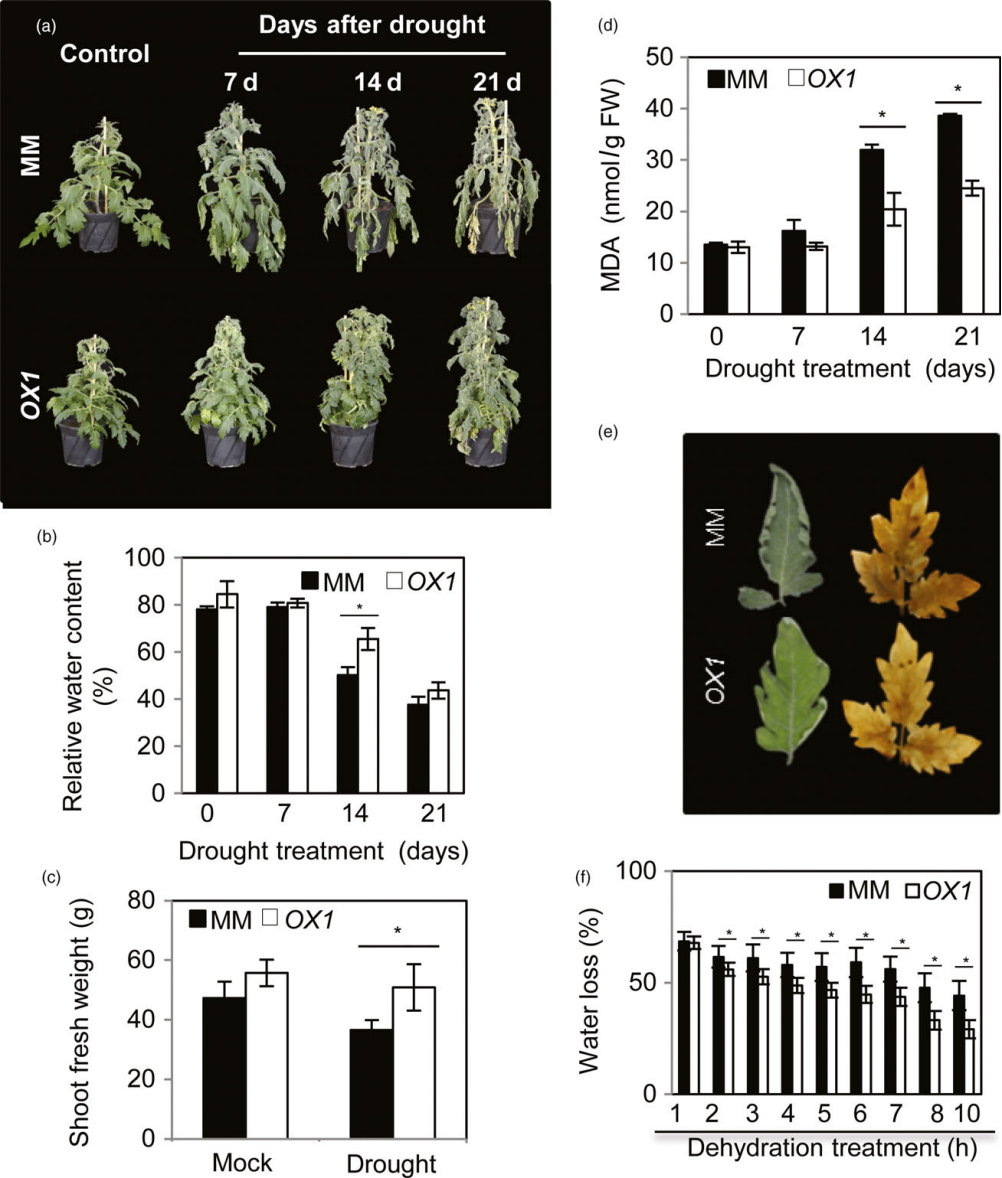
Ectopically expressed *AtJUB1‐GFP* in tomato confers tolerance to water deprivation. (a) Phenotype of *AtJUB1*‐expressing (*OX1*) and wild‐type tomato cv. Moneymaker (MM) plants under well‐watered control (left) and water‐deficit conditions (right): 42‐day‐old plants were subjected to drought for 7, 14 and 21 days. Note the more severe wilting in MM plants. (b) Relative water content of terminal leaflets (leaf no. 2) of MM and *OX1* plants measured during drought treatment. Data represent the means ± SD (*n* = 4 independent experiments). (c) Shoot fresh weight of MM and *OX1* plants after 21 days of drought. (d) Malondialdeyhde (MDA) content of MM and *OX1* plants during water deprivation. (e) Wilting phenotype and DAB staining for ROS accumulation in detached leaves of MM and *OX1* plants, 10 h after start of the dehydration treatment. (f) Percent water loss in detached leaves of MM and *OX1* plants. Data in (c), (d) and (f) represent the means ± SD (*n* = 3). Asterisks (*) indicate statistically significant differences between MM and *OX1* according to Student's *t*‐test (*P *<* *0.05).

Given the relationship between transpiration rate and the area, shape and surface characteristics of leaves (Alpert, 2006), only moderately overexpressing *AtJUB1* plants (*OX1* and *OX3*) were used for the analysis of drought responses in this study. To this end, 42‐day‐old *OX* and MM plants were subjected to water deprivation for up to 21 days. As shown in Figure 4a and Figure S2 *OX* plants exhibited higher tolerance to water‐deficit stress (delayed leaf wilting) than wild‐type plants (MM) at all indicated time points. Measurements of relative water content (RWC) in leaf tissues revealed no significant difference between *OX* and MM plants at control condition (0 day) and at early stage of drought (7 days), while at later stages of drought (14 days) higher RWC was observed in *OX* compared to MM plants (Figure 4b).

Shoot biomass after 21 days of water withholding was higher in *OX* than in MM plants (Figure 4c). The content of malondialdeyhde (MDA), a marker of lipid peroxidation, was drastically elevated in MM plants, but not in the *OX* plants, at the later stages of drought (14 and 21 days) (Figure 4d).

Next, we quantified the activities of several enzymatic antioxidants (ascorbate peroxidase, APX; peroxidase, POX; glutathione reductase, GR; monodehydroascorbate reductase, MDHAR; and dehydroascorbate reductase, DHAR) in MM and *OX* tomato plants under control (nonstress) and drought (7, 14 and 21 days) conditions. Of those, the activities of APX and POX increased with plant age (control condition) in MM, but no significant change was observed in *OX* plants (Figure S3). Activities of all measured enzymes increased with the progression of drought (after 14 and 21 days) in both genotypes; however, this induction was less dramatic in *OX* than in MM plants (Figure S3). This result is in accordance with higher H_2_O_2_ and MDA contents and thus the enhanced level of oxidative stress in MM plants at advanced stages of drought.

The response of plants to water deficit includes a reduction in transpiration and thus loss of water vapour from leaves. To analyse the rate of water loss in *OX* and MM plants, subterminal leaflets of leaf no. 2 were detached and analysed over a 10‐h period. Notably, while MM leaves showed extensive wilting after 10 h of desiccation, leaves of *OX* plants showed only slight wilting (Figure 4e). In accordance with this, the rate of water loss was higher in MM than in *OX* plants (Figure 4f). Consequently, a lower level of H_2_O_2_ was observed in detached leaves of *OX* after 10 h of desiccation (Figure 4e). Similar results were obtained when *OX* and MM plants at a younger developmental stage (21 days old) were subjected to water deprivation (Figure S4).

We also examined *AtJUB1* overexpression plants under polyethylene glycol (PEG)‐triggered water‐deficit condition. To this end, 42‐day‐old *OX* and MM plants were irrigated with 25% PEG 6000 for a period of 7 days, while irrigation with water was used in control experiments. As demonstrated in Figure S5, *AtJUB1* overexpressors better survive PEG irrigation, whereas MM plants show severe wilting and chlorosis after 7 days of PEG treatment. In accordance with this, MDA content was elevated (~threefold) in MM compared to *OX* plants. Taken together, our results strongly suggest that JUB1 functions as a positive regulator in the response to drought stress in tomato.

### AtJUB1 directly activates transcription of *SlDREB1, SlDREB2* and *SlDELLA* upon water deprivation

**Figure 5:**
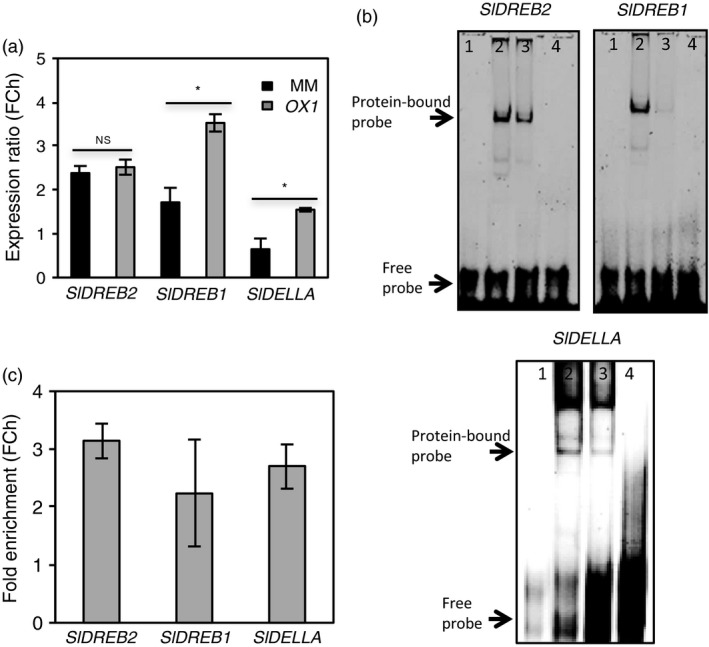
AtJUB1 directly regulates *SlDREB2, SlDREB1* and *SlDELLA*. (a) Expression of *SlDREB2*,* SlDREB1* and *SlDELLA* in MM and *AtJUB1‐GFP* (*OX1*) plants upon 7 days of withholding water. Expression was analysed by qRT‐PCR. Values were normalized to those determined in the well‐watered controls. Data represent the means ± SD (*n* = 3). Asterisks represent statistically significant differences between MM and *OX1* plants according to Student's *t*‐test (*P *<* *0.05). (b) EMSA showing binding of AtJUB1 to *SlDREB2, SlDREB1* and *SlDELLA* promoter regions harbouring the AtJUB1 binding site; 1, labelled probe (5′‐DY682‐labelled double‐stranded oligonucleotides) only; 2, labelled probe plus AtJUB1‐GST protein; 3, labelled probe, AtJUB1‐GST protein and 100× competitor (unlabelled oligonucleotide containing SlJUB1 binding site); 4, labelled probe plus GST protein. (c) ChIP‐qPCR shows enrichment of *SlDREB2, SlDREB1* and *SlDELLA* promoter regions containing the AtJUB1 binding site. For ChIP experiments, terminal leaflets (from leaf no. 2) of *AtJUB1‐GFP* tomato plants were harvested after drought treatment (7 days). Values were normalized to the values for *Solyc01G090460* (promoter lacking an AtJUB1 binding site). qPCR was used to quantify the enrichment of *SlDREB2, SlDREB1* and *SlDELLA* promoter regions. Data represent means ± SD (*n* = 3). FC, fold change.

To test whether AtJUB1 regulates drought stress by activating potential target genes of SlJUB1 (*SlDREB1*,* SlDREB2* and *SlDELLA*), we checked the expression of the three genes in *AtJUB1‐OX* and MM plants after 7 days of withholding water. Our results confirmed slight elevation of *SlDREB2* transcript abundance in *OX* compared to MM*,* but a significant induction in expression levels of *SlDREB1* and *SlDELLA* (Figure 5a). Next, we conducted EMSA experiments to dissect direct physical interaction between AtJUB1 and the promoter regions of the target genes. Results exhibit that AtJUB1, like SlJUB1, binds to the JUB1 binding motifs in the promoter regions of *SlDREB1*,* SlDREB2* and *SlDELLA*. Interaction appears to be specific, as retardation bands are abolished upon the addition of unlabelled promoter fragments (competitor) in excess (Figure 5b). Finally, we performed chromatin immunoprecipitation (ChIP) assays to determine the direct interaction between AtJUB1 and the promoters of *SlDREB1*,* SlDREB2* and *SlDELLA* upon drought stress (7 days) *in planta*. ChIP assay followed by qPCR revealed binding of AtJUB1 to the promoters of the three genes (Figure 5c) suggesting them as direct target genes of JUB1 during drought stress in tomato.

## Discussion

Transcription factor‐based engineering has been used as a powerful tool for improving stress tolerance in crops. Water deficit (drought) is one of the most adverse factors impacting plant growth and fitness. Several studies have shown that manipulation of drought‐responsive TFs can result in drought‐tolerant phenotypes in different crop species (reviewed by Rabara *et al*., 2014; Nakashima *et al*., 2014).

Tomato is a one the most important vegetable food crops worldwide. Although most tomato cultivars are drought sensitive, only few studies have so far been conducted to investigate the molecular regulatory networks involved in the response to water limitation in this plant. Transcriptome analyses have identified a number of TFs that are responsive to drought in tomato (Gong *et al*., 2010; Krasensky and Jonak, 2012). Functional analysis of such TFs and identifying their signalling pathways are important steps in elucidating drought response networks in tomato.

**Figure 6:**
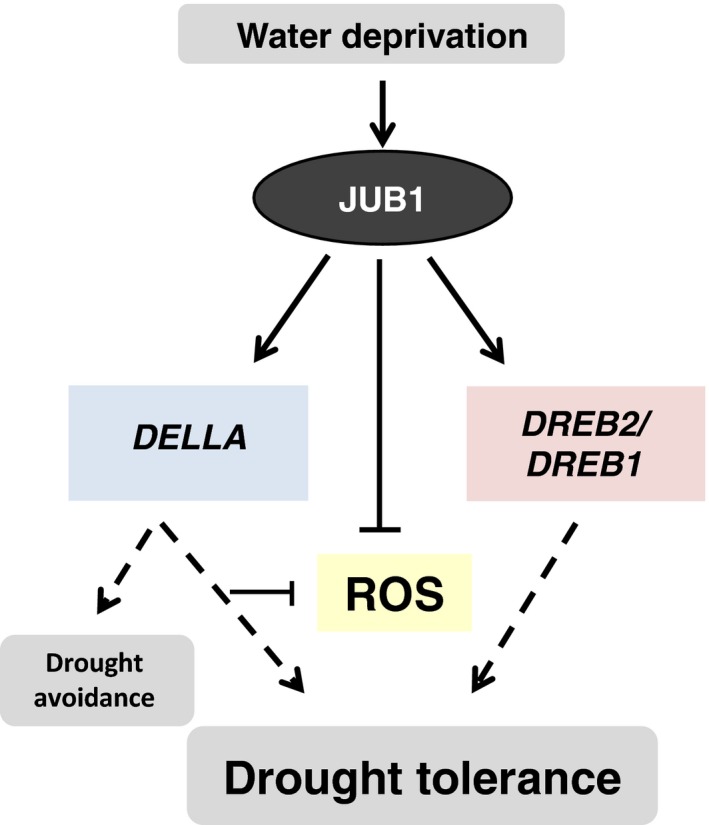
Model for the action of JUNGBRUNNEN1 (JUB1) in conferring tolerance to drought in tomato. Water deprivation triggers elevated expression of *SlJUB1*, which leads to activation of *DELLA* and the stress‐related genes *DREB2* and *DREB1*. This, together with reduced ROS levels, increases drought tolerance.

In this study, we identified SlJUB1, a homologue of the Arabidopsis NAC transcription factor JUNGBRUNNEN1 (JUB1), as a regulator of the response to drought stress in tomato and revealed its use as a tool to improve drought tolerance (Figure 6). *SlJUB1* expression is strongly induced upon treatment with H_2_O_2_, NaCl, PEG and dehydration, indicating a role for this TF in the regulation of abiotic stress response networks in tomato. Using a VIGS approach in *Solanum lycopersicum* cv. Moneymaker, we showed that a reduced level of *SlJUB1* impairs the water‐deficit response of both intact plants and detached leaves. Silencing of *SlJUB1* (*TRV2‐SlJUB1*) resulted in oxidative damage evidenced by accumulation of H_2_O_2_, and enhanced water loss under water‐limiting conditions (Figure 2d and f). Conversely, tomato plants ectopically and moderately expressing *AtJUB1* (*AtJUB1‐OX*) exhibited enhanced tolerance to water deficit without a significant penalty on growth. The intracellular level of H_2_O_2_ as well as water loss was significantly reduced during drought stress in tomato plants expressing *AtJUB1* (Figure 4e and f), indicating that JUB1 is a positive regulator of the response to drought in tomato.

We have previously shown that Arabidopsis JUB1 restricts plant growth and enhances tolerance to abiotic stresses by affecting multiple and interconnected cellular pathways involved in phytohormone biosynthesis/signalling and ROS signalling (Shahnejat‐Bushehri *et al*., 2016; Wu *et al*., 2012). AtJUB1 directly represses genes that are critical for GA and BR biosynthesis (*GA3ox1* and *DWF4*, respectively), while it directly activates the DELLA‐encoding genes *GAI* and *RGL1,* thereby leading to the accumulation of DELLA proteins. Furthermore, AtJUB1 directly targets and activates expression of *DREB2A*, a key transcription factor for the regulation of drought and heat responses in Arabidopsis (Kant *et al*., 2008; Sakuma *et al*., 2006; Wu *et al*., 2012).

Transcript analysis revealed that several drought‐responsive genes were differentially expressed between *TRV2‐SlJUB1*,* AtJUB1‐OX* and control tomato plants upon drought stress. Interestingly, among those, transcript levels of *SlDREB1* and *SlDREB2,* homologues of Arabidopsis *DREB2A,* and of *SlDELLA*, a homologue of Arabidopsis *GIBBERELLIC ACID INSENSITIVE* (*GAI*), were significantly reduced in *TRV2‐SlJUB1* (Figures 2g and 3c)*,* while *SlDREB1* and *SlDELLA* were enhanced in *AtJUB1‐OX* compared to control plants during drought stress (Figure 5a). However, transcript levels of GA and BR biosynthesis genes were not different between *TRV2‐SlJUB1* and *AtJUB1‐OX* plants during water deficit (data not shown).

By EMSA and ChIP, we demonstrated that AtJUB1 directly interacts with *SlDREB1*,* SlDREB2* and *SlDELLA* promoters and regulates their transcription *in planta* (Figure 5b,c). Furthermore, EMSA experiments revealed binding of tomato SlJUB1 to the promoters of *SlDREB1*,* SlDREB2* and *SlDELLA* raising the possibility that they may be direct SlJUB1 target genes (Figure 5b). Taken together, these results indicate that the function of JUB1 and the stress regulatory network controlled by this TF is considerably conserved between Arabidopsis and tomato.

*SlDREB1* and *SlDREB2,* the putative target genes of SlJUB1, are two homologues of DREB2‐type TFs in tomato. DREB (dehydration‐responsive element‐binding) proteins constitute a subfamily of the plant‐specific AP2/ERF TF family. They interact with DRE/CRT (dehydration‐responsive element/C‐repeat element) *cis*‐elements present in the promoters of target genes and regulate plant responses to diverse abiotic stresses, particularly cold and drought. *DREB* genes from several species have been reported to be functionally involved in the regulation of plant responses to drought. These include Arabidopsis *DREB1A* and *DREB2A* (Kudo *et al*., 2016; Yamaguchi‐Shinozaki and Shinozaki, 2006), soya bean *GmDREB2* (Chen *et al*., 2007) and *GmERF3* (Zhang *et al*., 2009), tomato *JERF1* (Zhang *et al*., 2010) and *SIERF5* (Pan *et al*., 2012) and apple *MsDREB6.2* (Liao *et al*., 2016), among others.

*SlDREB1* and *SlDREB2* are homologous to Arabidopsis *DREB2A* (53.1% and 44.9% similarity at the amino acid level), an important TF regulating drought responses (Yamaguchi‐Shinozaki and Shinozaki, 2006). *DREB2A* expression is negatively regulated by GROWTH‐REGULATING FACTOR 7 (GRF7), but positively regulated by JUB1 in Arabidopsis (Kim *et al*., 2011; Wu *et al*., 2012; Yoshida *et al*., 2014). Overexpression of constitutively active DREB2A resulted in growth retardation and significant drought stress tolerance (Sakuma *et al*., 2006). It has been shown that SlDREB2 also regulates general plant growth and the response to salinity stress. Overexpression of *SlDREB2* in tomato resulted in a semi‐dwarf phenotype associated with a reduced level of physiologically active gibberellic acids (GAs) (Hichri *et al*., 2016). *SlDREB2* overexpression significantly enhances tolerance to salinity by affecting multiple cellular processes such as, *inter alia*, enhanced synthesis of osmoprotectants, accumulation of abscisic acid (ABA) and the regulation of stress‐responsive genes (Hichri *et al*., 2016). However, the functions of *SlDREB1* and *SlDREB2* in the response to drought stress in tomato remain to be characterized, although the homology to *DREB2A* from Arabidopsis suggests similarity in function.

To respond to, and resist, water deficit, plants have evolved various strategies enabling them to integrate activities at the whole‐plant level. These strategies may involve drought avoidance and/or the development of drought tolerance mechanisms. Drought avoidance is accompanied by changes in organ morphology such as a reduction in leaf area which may be accompanied by stomatal closure as well as changes in root thickness or length (Anjum *et al*., 2011; Reddy *et al*., 2004), whereas drought tolerance includes maintaining cell turgor and reducing evaporative water loss by accumulating compatible solutes without disruption of cellular metabolism (Munns, 1988; Price *et al*., 2002; Savé *et al*., 1993; Yancey *et al*., 1982). Constitutively high overexpression of *AtJUB1* in both Arabidopsis and tomato results in significant growth reduction and reduced leaf area as a consequence of GA and brassinosteroid (BR) deficiencies (Shahnejat‐Bushehri *et al*., 2016, 2017). These plants are expected to better ‘avoid’ drought, for example through the reduced evaporative leaf surface. To test whether JUB1 is also involved in the regulation of drought tolerance, we analysed tomato plants moderately expressing *AtJUB1* (*OX1* and *OX3*) which only marginally affects growth. The plants showed significantly higher survival than wild type and less water loss during drought, suggesting that in addition to the reduction in growth and leaf surface area, JUB1 employs other mechanisms to allow plants to cope with water deficit. However, by analysing nail polish imprints of the abaxial leaf surface, we did not observe an obvious difference in stomatal aperture between wild‐type and *AtJUB1* overexpression plants and primary root length was also not significantly different between both types of plants during drought (data not shown).

Accumulation of reactive oxygen species (ROS) due to enhanced ROS production and/or reduced ROS scavenging capacity are the inevitable consequences of drought stress. Although ROS can act as signal molecules, elevated levels of ROS cause oxidative damage to essentially all cellular components including membranes, proteins and nucleic acids, thereby causing metabolic dysfunction and cell death. Manipulating ROS levels may thus represent a promising strategy to improve stress tolerances of crop plants under a variety of unfavourable environmental conditions. Indeed, it has been demonstrated that several genes, including TFs, mediate abiotic stress resistance through the regulation of cellular ROS levels (reviewed by You and Chan, 2015). Here, we demonstrated that decreased expression of *SlJUB1* (in VIGS‐silenced plants) increased the accumulation of H_2_O_2_ and, accordingly, resulted in oxidative damage of cell membranes and reduced tolerance to drought stress. In contrast, heterologous expression of *AtJUB1* lowered H_2_O_2_ level, resulting in enhanced tolerance to drought stress. A similar function has recently been reported for *JUB1* in banana (*Musa acuminata*). Banana plants overexpressing *MusaNAC042* (the closest homologue of *AtJUB1* in this species) exhibit significantly reduced stress‐induced oxidative damage evidenced by a lower level of MDA (as observed here for *AtJUB1* overexpressing tomato plants) and a higher photosynthetic activity. In addition, proline, a likely important osmoprotectant in plants (Ashrafa and Fooladb, 2007), accumulated in *MusaNAC042* overexpressors compared to control plants, concomitant with an improved tolerance to drought and high salinity stress (Tak *et al*., 2016). Of note, elevated levels of proline, in addition to the osmoprotectant trehalose, were also detected in tissues of Arabidopsis and tomato *AtJUB1* overexpressors (Shahnejat‐Bushehri *et al*., 2017; Wu *et al*., 2012), suggesting that compatible solute osmolytes contribute to the enhanced drought tolerance in these plants in accordance with the important role of osmotic adjustment during drought conditions (Blum, 2017).

In Arabidopsis, JUB1 has been shown to dampen the intracellular level of H_2_O_2_ via direct activation of *DREB2A*, which in a regulatory cascade is upstream of genes encoding several HSPs and H_2_O_2_ scavenging enzymes, as well as through a direct activation of DELLA‐encoding genes thereby triggering the accumulation of DELLA proteins. In Arabidopsis, it has been demonstrated that DELLA proteins restrain plant growth and promote survival under abiotic stress conditions via an enhancement of ROS scavenging capacity (Achard *et al*., 2008b). Similar to Arabidopsis, our data reported here suggest that reduced levels of H_2_O_2_ are likely due to the activation of *SlDREB1, SlDREB2* and *SlDELLA* by SlJUB1 in tomato.

Collectively, our findings and those reported by Tak *et al*. (2016) on banana suggest that JUNGBRUNNEN1 can be employed to enhance drought tolerance in both dicot and monocot species including crops. The potential effect of *JUB1* expression on organ growth (Shahnejat‐Bushehri *et al*., 2016) can be avoided by selecting lines expressing *JUB1* at a moderate level (as we did here for tomato), or by driving expression of *JUB1* from abiotic stress‐inducible promoters such as previously shown for *AtJUB1* in Arabidopsis (Shahnejat‐Bushehri *et al*., 2016; Wu *et al*., 2012).

## Experimental procedures

### General

Oligonucleotides (Table S1) were obtained from Eurofins MWG Operon (Ebersberg, Germany). PLAZA 3.0 (http://bioinformatics.psb.ugent.be/plaza/; Proost *et al*., 2015) and the Sol Genomics webpage (https://solgenomics.net/) were employed for the identification of tomato orthologs.

### Plant materials, growth conditions

*Solanum lycopersicum* L. cv. Moneymaker (MM) was used as the wild type (control) in all experiments. To generate lines overexpressing *AtJUB1*, tomato plants were transformed with the *AtJUB1‐GFP* overexpression construct (Wu *et al*., 2012). Seeds were germinated on Murashige–Skoog (MS) medium containing 2% (w/v) sucrose and then transferred to soil containing a mixture of potting soil and quartz sand (2:1, v/v) and grown in a growth chamber at 500 μmol photons/m^2^/s and 25 °C under a 14/10‐h light/dark regime as described previously (Schauer *et al*., 2005). All plants were watered in the same way using a drip irrigation system.

### Stress treatments

For stress treatments, seeds were germinated on full‐strength MS medium containing 2% (w/v) sucrose and grown under a 16‐h light (23 °C)/8‐h dark (20 °C) regime. Three‐week‐old uniformly sized seedlings were transferred to liquid MS medium in flasks containing polyethylene glycol (20% [w/v] PEG 6000), H_2_O_2_ (5 mm) or NaCl (200 mm). For desiccation, terminal leaflets (from leaf no. 2) were detached and subjected to air‐drying for the indicated time points.

Tomato plants grown in soil were well irrigated for 42 days after germination (DAG) before stress treatments. In each experiment, fifteen plants per genotype were used for the treatments. Drought stress was induced by withholding water for up to 21 days. PEG‐mediated drought stress was applied by irrigating plants with 25% (w/v) PEG 6000‐water for 7 days. Control plants were well watered throughout the experiment. All stress treatment experiments were performed in three independent trials.

### Virus‐induced gene silencing

VIGS (virus‐induced gene silencing) was performed using VIGS vectors *pTRV1* (*pYL192*) and *pTRV2* (*pYL170*) (Liu *et al*., 2002). *pTRV2‐attL2‐SlJUB1‐attL1:* the *SlJUB1* coding sequence was amplified by PCR from *Solanum lycopersicum* cv. Moneymaker leaf cDNA and cloned into pENTR‐D‐TOPO using pENTR Directional TOPO Cloning kit (Invitrogen, Karlsruhe, Germany). Primer sequences are given in Table S1. The sequence‐verified entry clone was then recombined into *pTRV2* by LR recombination using LR reaction mix II (Life Technologies, Karlsruhe, Germany). *pTRV1* and *pTRV2‐attL2‐SlJUB1‐attL1* were transformed into *Agrobacterium tumefaciens* (strain GV3101) and subsequently used for the infection of tomato seedlings (Senthil‐Kumar and Mysore, 2014).

### Subcellular localization of SlJUB1

The *SlJUB1* coding sequence (without stop codon) was amplified by PCR from *S. lycopersicum cv*. Moneymaker leaf cDNA (primers listed in Table S1) and cloned into pDONR 207 using BP clonase (Invitrogen). The sequence‐confirmed entry vector was recombined into pK7FWG2 (Karimi *et al*., 2002) using LR reaction mix II (Life Technologies). The recombined plasmid (*35S:SlJUB1‐GFP*) was transformed into *Agrobacterium tumefaciens* (strain GV3101) and then used for infiltration of *N. benthamiana* leaves (Senthil‐Kumar and Mysore, 2014). GFP signal was analysed using a Leica DM6000B/SP5 confocal laser scanning microscope (Leica Microsystems, Wetzlar, Germany).

### Relative water content

Relative water content of leaves was determined as described (Wang *et al*., 2015). Briefly, terminal leaflets of leaf no. 2 were harvested and weighed immediately to determine their fresh weight (FW). Subsequently, leaves were immersed in distilled water and incubated at 4 °C overnight to obtain the saturated weight (SW). Leaves were then dried at 60 °C for 48 h to measure the dry weight (DW). RWC was calculated using the formula RWC % = ((FW−DW)/(SW‐DW)) × 100.

### Water loss

Water loss was determined as reported (Raineri *et al*., 2015). In brief, detached terminal leaflets (leaf no. 2) were placed in a growth chamber at 20–22 °C. Leaf weight was recorded at time points indicated in the figures and expressed as percentage of the initial fresh weight.

### Ion leakage

Terminal leaflets of leaf no. 2 were immersed in 40 mL deionized water and shaken at room temperature for 8 h. Initial electrical conductivity was measured at 25 °C using a conductometer (SI Analytics, Mainz, Germany). Thereafter, samples were boiled at 100 °C for 30 min and left at room temperature until 25 °C was reached, and total conductivity was measured again. Ion leakage is expressed as the percentage of initial conductivity of the total conductivity; low and high percentage values indicate little or strong membrane damage, respectively.

### Quantification of MDA and 3,3′‐diaminobenzidine staining

Lipid peroxidation was assessed by measuring malondialdeyhde (MDA) levels (Hodges *et al*., 1999). 3,3′‐Diaminobenzidine (DAB) was used as an indicator of H_2_O_2_ levels (Fryer *et al*., 2002).

### Enzyme measurements

Samples were ground to a fine powder in liquid nitrogen and 0.1 g powder was homogenized in 500 μL of 50 mm Tris–HCl, pH 7.8, containing 0.1 mm EDTA, 0.1% (w/v) Triton X‐100 and 1% (w/v) polyvinylpolypyrrolidone (PVPP). For the determination of APX activity, 5 mm ascorbate was added. Samples were centrifuged at 10 000 *g* for 10 min, and supernatants were used for measurements. Spectrophotometric analyses were conducted using a Shimadzu UV‐1700 spectrophotometer.

POX (EC 1.11.1.7) activity was determined following the method of Herzog and Fahimi (1973). The increase in the absorbance at 465 nm due to oxidation of diaminobenzidine (DAB) was followed for 1 min. One unit of POX activity was defined as 1 μmol H_2_O_2_ decomposed in 1 min. GR (EC 1.6.4.2) activity was measured according to Foyer and Halliwell (1976). Activity was calculated using the extinction coefficient of NADPH at 340 nm (6.2 mm ^−1^ cm^−1^). One unit of GR was defined as 1 μmol GSSG reduced in 1 min. APX (EC 1.11.1.11) activity was measured according to Nakano and Asada (1981). The assay depends on the decrease in absorbance at 290 nm as ascorbate is oxidized. MDHAR (EC 1.6.5.4) activity was determined according to Arrigoni *et al*. (1981); NADH oxidation by MDHAR was observed in the presence of ascorbate oxidase (1 U) at 340 nm. DHAR (EC 1.8.5.1) was measured based on the method described by Nakano and Asada (1981). The increase in the absorbance at 265 nm was recorded.

### Expression profiling

Total RNA extraction, cDNA synthesis and qRT‐PCR were performed as described (Balazadeh *et al*., 2008). Arabidopsis drought marker genes were extracted from the literature (Sakuraba *et al*., 2015) and in‐house experiments. ROS‐responsive genes were extracted from Gechev *et al*. (2004), Gechev and Hille (2005) and Wu *et al*. (2012). Tomato orthologs of the Arabidopsis genes were identified using PLAZA 3.0 and annotated using the Sol Genomics database. qRT‐PCR primers (Table S1) were designed using QuantPrime (Arvidsson *et al*., 2008). PCRs were run on an ABI‐PRISM 7900 HT sequence detection system (Applied Biosystems, Darmstadt, Germany), and amplification products were visualized using SYBR Green (Applied Biosystems). *SlGAPDH (GLYCERALDEHYDE PHOSPHATE DEHYDROGENASE; Solyc04g009030*) served as reference gene for data analysis.

### Chromatin immunoprecipitation

Tomato leaves expressing AtJUB1‐GFP protein under the control of the cauliflower mosaic virus (CaMV) 35S promoter were used to perform chromatin immunoprecipitation (ChIP). ChIP was performed according to Kaufmann *et al*. (2010). Primers used to amplify the promoter regions of *SlDREB1*,* SlDREB2* and *SlDELLA* containing JUB1 binding sites are listed in Table S1. Primers annealing to the promoter of gene *Solyc01G090460*, lacking a JUB1 binding site, were used as negative control.

### Electrophoretic mobility shift essay

Recombinant AtJUB1‐GST, SlJUB1‐GST and GST proteins were prepared as described (Puranik *et al*., 2011). *AtJUB1* and *SlJUB1* coding sequences were PCR‐amplified from Arabidopsis or tomato cDNA, respectively, using primers listed in Table S1. PCR products were GATEWAY‐recombined into pDEST24 destination vector (Invitrogen). Recombinant vectors were transformed into *Escherichia coli* Star (DE3) pRARE generated by transforming the pRARE plasmid isolated from Rosetta (DE3) pRARE cells (Merck, Darmstadt, Germany) into *E. coli* BL21 Star (DE3) (Invitrogen). Recombinant GST fusion proteins were purified using glutathione agarose beads (Sigma‐Aldrich, Taufkirchen, Germany). 5′‐DY682‐labelled oligonucleotides, purchased from Eurofins MWG Operon, were annealed to form the probe DNA. EMSA reactions were performed using the Odyssey Infrared EMSA kit (LI‐COR, Bad Homburg, Germany).

### Statistical analysis

Statistical analysis of the bioassays was performed using GraphPad Prism (GraphPad Software Inc., San Diego, CA). Experimental data were analysed with Student's *t*‐test at *P < *0.05.

### Multiple sequence alignment

Multiple sequence alignment of SlJUB1 with other known NAC proteins was carried out using Clustal Omega (Sievers *et al*., 2011).

## Funding

This work was supported by funding from the Deutsche Forschungsgemeinschaft (DFG) to S.B. (BA 4769/2‐1). Financial support was furthermore provided by the University of Potsdam and the MPI of Molecular Plant Physiology.

## Competing interests

The authors declare no competing financial interests.

## Supporting information


**Figure S1** Amino acid sequence alignment of SlJUB1 with other known NAC transcription factors.
**Figure S2** Phenotypes of *AtJUB1* expressing (*OX3*) and wild‐type tomato cv. Moneymaker (MM) plants under drought stress.
**Figure S3** Lower ROS scavenging enzyme activities in tomato plants ectopically expressing *AtJUB1* (*OX1*).
**Figure S4** Ectopic expression of *AtJUB1‐GFP* in tomato confers tolerance to water deficit in younger plants.
**Figure S5** Tomato plants ectopically expressing *AtJUB1* show enhanced tolerance to exogenous treatment with polyethylene glycol.

**Table S1** Oligonucleotide sequences.
