# Supplementary material for: NAC transcription factor JUNGBRUNNEN1 enhances drought tolerance in tomato
Source: Plant Biotechnol J. 2017 Aug 4;16(2):354–66. doi: 10.1111/pbi.12776 (PMC5787828; doi:10.1111/pbi.12776)
Supplement: Supplementary file 1 — Figure S1 Amino acid sequence alignment of SlJUB1 with other known NAC transcription factors. Figure S2 Phenotypes of AtJUB1 expressing (OX3) and wild‐type tomato cv. Moneymaker (MM) plants under drought stress. Figure S3 Lower ROS scavenging enzyme activities in tomato plants ectopically expressing AtJUB1 (OX1). Figure S4 Ectopic expression of AtJUB1‐GFP in tomato confers tolerance to water deficit in younger plants. Figure S5 Tomato plants ectopically expressing AtJUB1 show enhanced tolerance to exogenous treatment with polyethylene glycol. [file PBI-16-354-s001.pdf]

**Figure S1**

```
                                 A                                  B
AtJUB1 MSGEGNLGKDHEEENEAPLPGFRFHPTDEELLGYYLRRKVENKTIKLELIKQIDIYKYDP
SlJUB1 --MVDEIKTMSKEEEDVILPGFRFHPTDEELIGFYLRRKVENKRISMELIRHIDIYKYDP
SlNAM  --MEIYHQMQFDCGDPHLPPGFRFHPTDEELITYYLLKKVLDCNFTARAIAEVDLNKCEP
SlNAC1 -----MNKGANGNQQLELPAGFRFHPTDDELVQHYLCRKCAGQSIAVSIIAEIDLYKFDP
SlNAC2 ---------MVGKISSDLPPGFRFHPTDEELIMYYLRYQATSRPCPVSIIPEIDVYKFDP
SlNAC3 --MESTDSSTGSHHQPQLPPGFRFHPTDEELVVHYLKKRVASVPLPVSIIAEVDLYKFDP
                   .        ********:**: .**   :  .       *  .:*: *  :*

         B                   C                                  D
AtJUB1 WDLPRVSSVGEKEWYFFCMRGRKYRNSVRPNRVTGSGFWKATGIDKPVYSNLD---CVGL
SlJUB1 WDLPKA---DDKEMYYFCMRGRKYKNSVRPNRVTKGGFWKATGIDKPVYSSTTKCIIIGL
SlNAM  WELPGKAKMGEKEWYFFSLRDRKYPTGLRTNRATEAGYWKATGKDREIFSSK-TCALVGM
SlNAC1 WQLPEKALYGEKEWYFFSPRDRKYPNGSRPNRAAGTGYWKATGADKPV---G-KPKTLGI
SlNAC2 WVLPEKAEFGDNEWYFFTPRDRKYPNGVRPNRAAVSGYWKATGTDKAIYSA---NKYVGI
SlNAC3 WELPAKATFGEQEWYFFSPRDRKYPNGARPNRAATSGYWKATGTDKPVLTAG-GTQKVGV
       * **      .::* *:*  *.***  .. * **.:  *:***** *: :         :*:

                          NLS
                  D                                         E
AtJUB1 KKSLVYYLGSAGKGTKTDWMMHEFRLPSTTKTD---------SPAQQAEVWTLCRIFKRV
SlJUB1 KKCLVYYRGSAGKGTKTDWMMHEFRLPPTSNT------------NQQAEVWTLCRIFKRS
SlNAM  KKTLVFYRGRAPKGEKSNWVMHEYRLDGKFAYH--------YISRSSKDEWVISRVFQKS
SlNAC1 KKALVFYAGKAPRGIKTNWIMHEYRLANVDRSA-------GKNNNLRLDDWVLCRIYNKK
SlNAC2 KKALVFYKGKPPKGVKTDWIMHEYRLSDSKSQTS------KQSGSMRLDDWVLCRIYKKK
SlNAC3 KKALVFYGGKPPKGVKTNWIMHEYRLADNKTNNKPPGCDLANKKSLRLDDWVLCRIYKKN
       ** **:* *   :* *::*:***:**                        : *.:.*::::

AtJUB1 TSQRNPTILPPNRKPVITLTDTCSKTSSLDSDHTSHRT--VDSMSHEPPL------PQP-
SlJUB1 SSTYTRSSYNTQE-VKHPIGDASSKACSLESDENSTDHDHSNSFRD-MSF------PHQ-
SlNAM  TGSNGAATSTGGGKKRL--SSSI-----NM----YQEVSSPSSVSHLPPLLDSSPYSTTA
SlNAC1 GTLEKHYNV--DNKET----TSF-----GEFDEEIKPKILPTQLAPMPPR--PRSTPAND
SlNAC2 NLGRTIEMMKVEEEELE--AQNV-----STTNNEIEVVGGPQTMK-LPRICSLSHLLELD
SlNAC3 NTQRPIDHERDD---LN--IDM------MMGSSSIHPSCIPNSMS-MPNIFGQPKIPQLK
                                                    .

AtJUB1 ----------------------------------------QNPYWNQHIVGFN----QPT
SlJUB1 ---------------------------------------NYNNYVAQMDETRNQQNPIPH
SlNAM  TS-AAAIVIGDRDRDHSF--KKEH--VPCFSTTATATITAQSLTFDPT----------SV
SlNAC1 YFYFESSESMTRMHTTNSSSGSEHVLSPCDKEVQS------APKWDEDHR--------NT
SlNAC2 --YFGSI-PQL-LSDN--------------------------LLYDDQGYTMNNVNNTSN
SlNAC3 SSNFGTTLIHD-QNDQNLYEGGSQY---SSKRPLA------NLYWNDQDGGASNDNSQST

AtJUB1 YTGNDNNLLMSFWNGN-----GGD----FI--GDSASWDELRSVIDGNTKP---------
SlJUB1 ESTFPSSSSISFWNTN-----STQEDYLFG--DHGNKWDDLKSIVDLATMM*--------
SlNAM  FDISSN-TLHALQPTPSFASILDSSPSNFTNYTRNSTFPSLRSLHENLQLPLFSGGTSAM
SlNAC1 LDFQLN-YLDGLLNEP-FETQMQQQICNFDQ------FNNFQDMFLYM-----QKPY---
SlNAC2 VDQVSS-QQQNTNN---I----TSNNCNIF--------FN-------YQQPLFVNPTFQ-
SlNAC3 KRFLTE-NMEDGLN---MNARADEQNGSIVS------LLSQQQVLGSLSEGVFRQPYSG-
                                                .

AtJUB1 ------------------------------
SlJUB1 ------------------------------
SlNAM  HGGFSNPMVNWTVPETQKVEQSELDCMWSY
SlNAC1 ------------------------------
SlNAC2 --------SQ--------------------
SlNAC3 --------MNWYS-----------------
```

**Figure S1** Amino acid sequence alignment of SlJUB1 with other known NAC transcription factors.

Gene codes and corresponding references for the known NAC proteins are as follows: AtJUB1, At2g43000 (Wu *et al*., 2012), SlNAM, Solyc07g062840 (Blein *et al*., 2008), SlNAC1, Solyc04g009440 (Selth *et al*., 2005), SlNAC2, Solyc05g007770 (Uppalapati *et al*., 2008), and SlNAC3, Solyc07g063420; Han *et al*., 2012). Note the highly conserved N-terminal DNA-binding domain with its motifs A – E (Zhu *et al*., 2014b). Fully conserved amino acid residues, conservative and semi-conservative amino acid exchanges are indicated by ´*´, ´:´ and ´.´, respectively. NLS, nuclear localization signal.

Figure S2

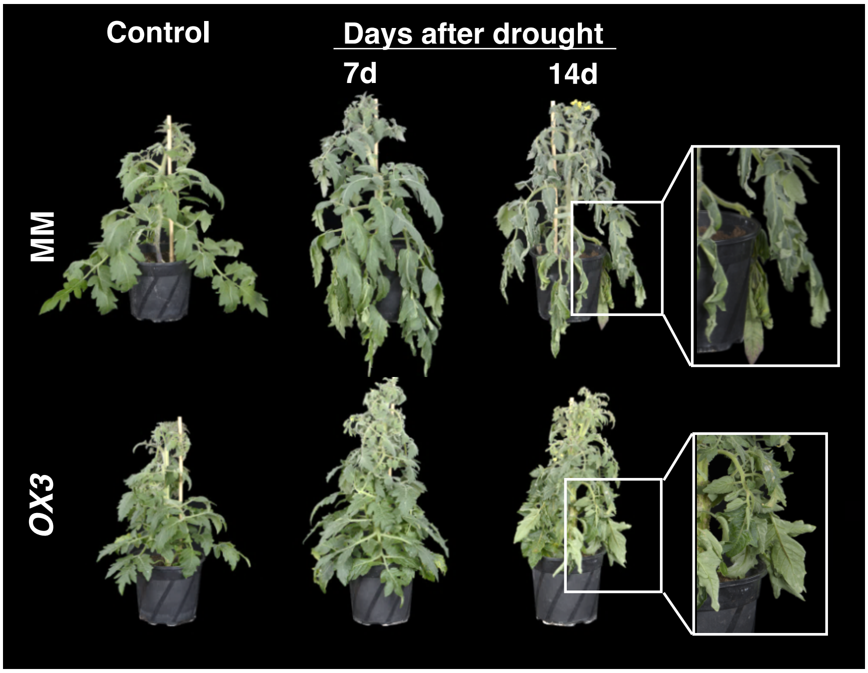


**Figure S2** Phenotypes of *AtJUB1* expressing (*OX3*) and wild-type tomato cv. Moneymaker (MM) plants under drought stress.

Plants were grown for 42 days in well-watered condition (left) and then subjected to drought for 7 days (middle) or 14 days (right). Note the less severe drought stress phenotype of *AtJUB1* expressing plants compared to MM.

**Figure S3**

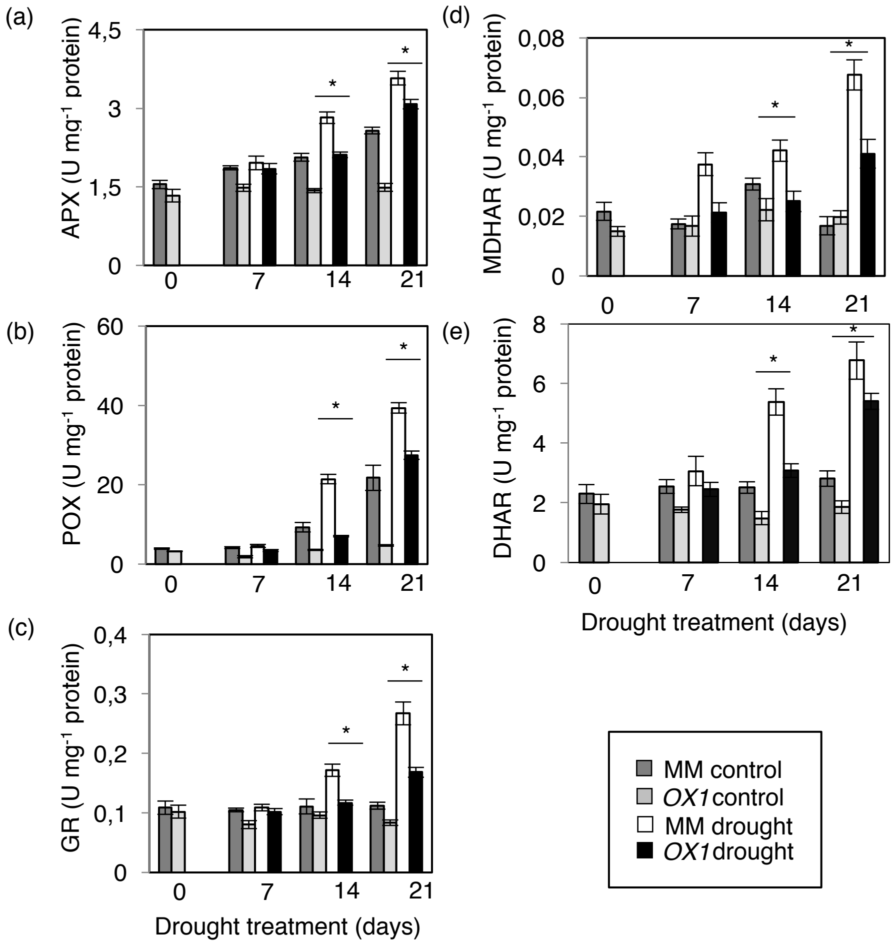


**Figure S3** Lower ROS scavenging enzyme activities in tomato plants ectopically expressing *AtJUB1* (*OX1*).

Activities of (a) ascorbate peroxidase (APX), (b) peroxidase (POX), (c) glutathione reductase (GR), (d) monodehydroascorbate reductase (MDHAR), and (e) dehydroascorbate reductase (DHAR) were determined in terminal leaflets of leaves no. 2 and 3 of MM and *OX* plants under control and water deprivation (7, 14 and 21 days) conditions. Data represent the means ± SD (n = 3 independent replicates). Asterisks represent statistically significant differences between MM and *OX* according to Student´s *t*-test ($p < 0.05$).

**Figure S4**

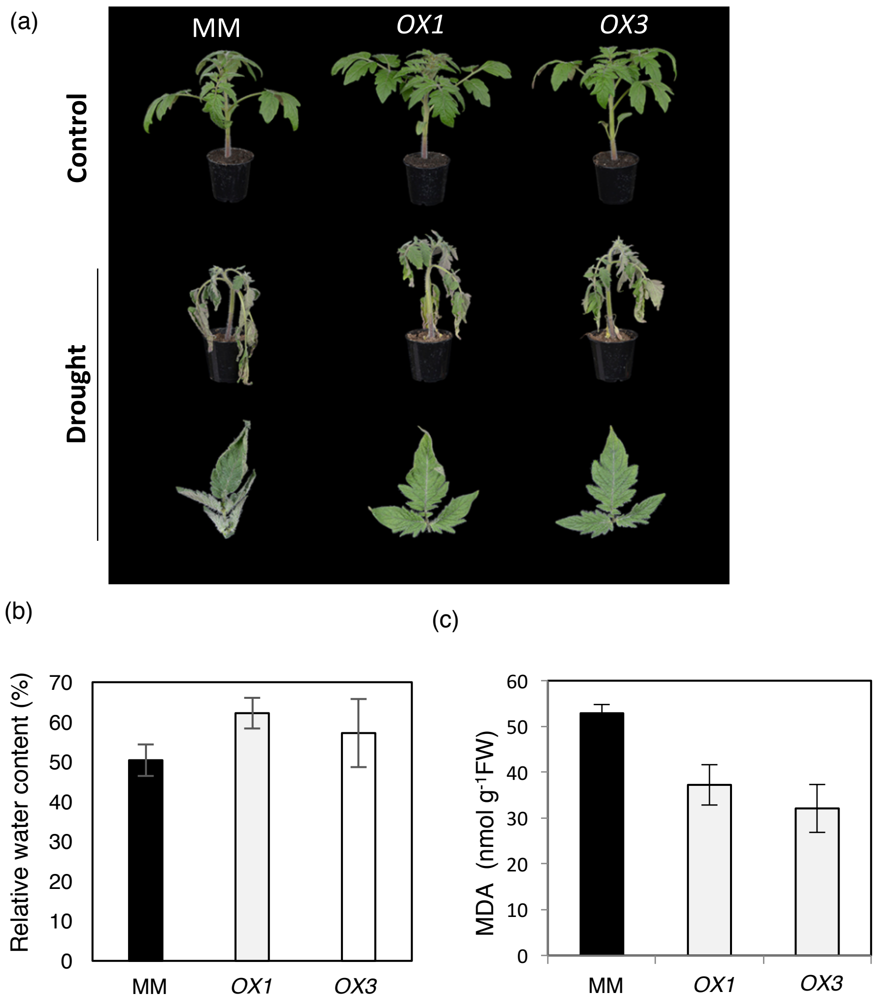


**Figure S4** Ectopic expression of *AtJUB1-GFP* in tomato confers tolerance to water deficit in younger plants.
(a) Top: Phenotype of 21-day-old wild-type tomato cv. Moneymaker (MM) and *AtJUB1* expressing (*OX1* and *OX3*) plants under well-watered control condition. Middle: Plants grown for 21 days under well-watered condition and then subjected to drought stress for 5 days. Bottom: Young leaves obtained from plants shown above. Note the better performance of *AtJUB1*-expressing lines during drought stress. (b) Relative water content of terminal leaflets (from leaf no. 3) of MM, *OX1* and *OX3* plants measured after 5 days of drought stress. (c) Malondialdeyhde (MDA) content of terminal leaflets of MM, *OX1* and *OX3* plants measured after 5 days of drought stress. Data in (b) and (c) represent means ± SD (n = 3).

**Figure S5**

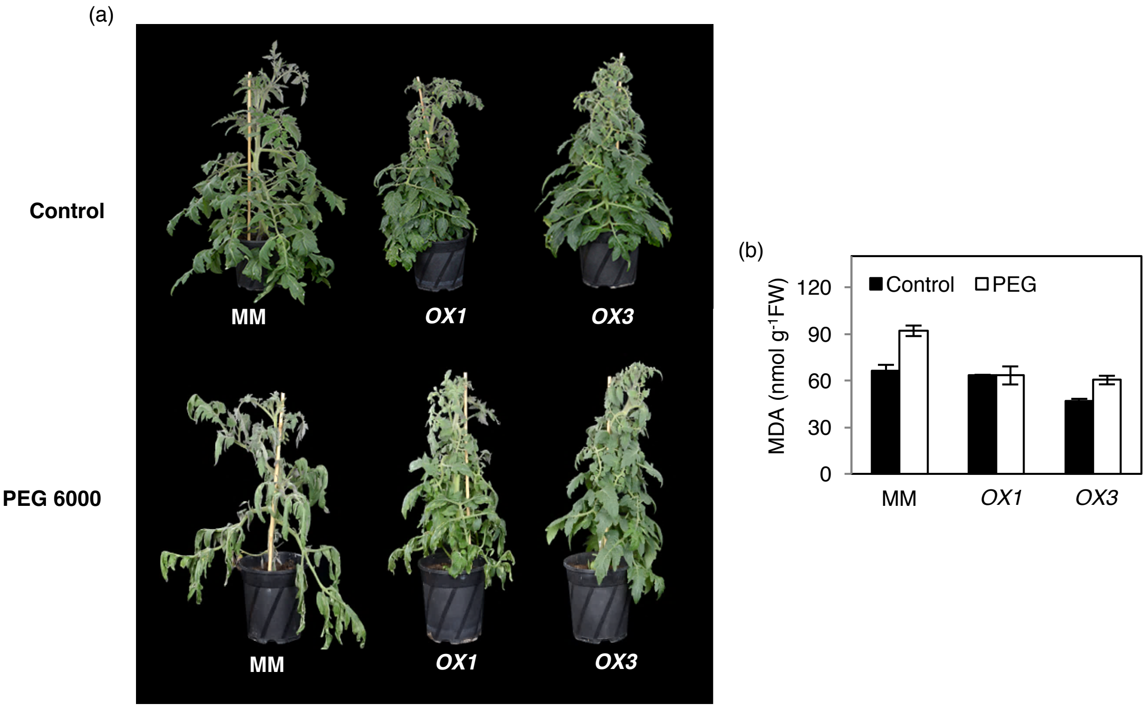


**Figure S5** Tomato plants ectopically expressing *AtJUB1* show enhanced tolerance to exogenous treatment with polyethylene glycol.

(a) Phenotypes of *AtJUB1* expressing (*OX1* and *OX3*) and wild-type tomato plants (cv. Moneymaker, MM) under control condition (upper row) and after treatment with PEG 6000 (lower row). For PEG treatment, 42-day-old plants were irrigated with 25% (w/v) PEG 6000 for 7 days. (b) MDA content in terminal leaflets (of leaf no. 2) of MM and *OX* plants under control condition and after irrigation with PEG 6000 for 7 days, as in (b). Data represent means ± SD (n = 3).
